# Supplementary material for: The Effect of Prolactin on Gene Expression and the Secretion of Reproductive Hormones in Ewes during the Estrus Cycle
Source: Animals (Basel). 2024 Jun 25;14(13):1873. doi: 10.3390/ani14131873 (PMC11240556; doi:10.3390/ani14131873)
Supplement: Supplementary file 1 [file animals-14-01873-s001.zip › animals-3049828-supplementary.pdf]

Table S1. Ingredients and nutrient composition of the basal diet (dry matter basis).

Item	Content (%)
Ingredient	
hay	42.00
mineral meal	0.40
Soybean meal	12.00
Corn	44.20
$CaHPO_4$	0.28
Premix[1)]	0.59
NaCl	0.53
Total	100.00
Nutritional Indicator	
Metabolic Energy, ME/ (MJ kg^{-1})[2)]	11.78
Crude Protein, CP	14.70
Ca	0.52
P	0.32

1) Provided per kilogram of Premix: VA:10260 IU, VE: 30IU, VD3: 2200 IU, Fe: 57.86mg, Zn: 42.73 mg, Mn: 33.65 mg, Cu: 9.34 mg, Se: 0.19 mg, I: 0.76 mg, Co: 0.23 mg.
